# Perceived social and media influences on tobacco use among Samoan youth

**DOI:** 10.1186/1471-2458-14-1100

**Published:** 2014-10-23

**Authors:** Judith McCool, Becky Freeman, Helen Tanielu

**Affiliations:** School of Population Health, Faculty of Medical and Health Science University of Auckland, Auckland, New Zealand; School of Public Health, University of Sydney, Sydney, Australia

**Keywords:** Family, Social structure, Media, Youth, Tobacco marketing, Samoa

## Abstract

**Background:**

Tobacco use among young Pacific populations continues to undermine efforts to reduce the escalating rates of non-communicable disease in the region. Reducing tobacco use to less than 5 percent by 2025 is now a World Health Organisation (WHO) mandated target for the Pacific region. Yet, little is known about the drivers to uptake of tobacco use among young people in the Pacific. Family and peers are expected to be important in this process, but similarly, tobacco marketing may also play an important role. The tobacco industry has been highly adaptive to the changing media environment across the Pacific Islands. The aim of this study was to develop an understanding of the social cultural and media drivers to tobacco uptake and use among young Samoans to contribute to the design of effective tobacco control intervention.

**Methods:**

We examined high school students (aged 16 and 17 years) perceptions of tobacco use in their community, access and use of media channels and the extent to which they are cognizant of both pro and anti-tobacco imagery across a range of media. Data was collected through in-depth semi-structured interviews. A thematic analysis of the interview data identified common and divergent beliefs, attitudes and behaviours surrounding tobacco use and the influence of the media.

**Results:**

Family is critically important for representing normative tobacco use in Samoa. The use of media, in particular digital media, was found to be conditioned by parental views on the use of media in the home. Media access remains highly regulated within more traditional households. Loyalty to traditional cultural practices (Fa’a Samoa) underpinned views on the limited influence of media on social norms around tobacco use. Parents were thought to have the greatest influence on youth smoking. Tobacco use was viewed as a personal, or family issue, and not a problem that was amendable to change at a societal level.

**Conclusion:**

In order to develop effective and culturally relevant tobacco control policies, the public health community must consider social norms around tobacco use as well as patterns of media use among young Samoans.

## Background

Tobacco use is the single most preventable cause of non-communicable disease (NCDs) globally. An agreed target for the Western Pacific region of a 10% reduction in current tobacco use (on baseline levels) by 2014, [1] and legislation to support reductions in tobacco use is now in place in many Pacific nations. Approximately 30% of the world’s smokers reside in the Western Pacific Region [WPR], where it is estimated that two people die every minute from diseases related to tobacco consumption; approximately 20% of all deaths for the region [2]. Although the WPR includes large tobacco consuming countries, such as China and Japan, estimates of current smokers in the small Pacific Islands countries and territories (PICTs) range between 17% and 49% [3]. Tobacco use in the PICTs is a significant contributor to the growing burden of NCD and consistent trends in other low and middle income regions, NCD prevalence is expected to escalate [4]. Without substantial collective effort, the NCD crisis facing the Pacific region will undermine the broader health agenda. Reducing tobacco use among young people is an essential component of a collective effort to reduce tobacco consumption in Samoa, and in other PICTs [4].

The Apia Communique, produced following the 2013 Pacific Health Ministers Meeting [5] resolved to take a strong, coordinated response to tobacco control in alignment with the WHO Framework Convention for Tobacco Control (FCTC) and the MPOWER framework [6, 7]. It is argued therefore that to reduce tobacco uptake, efforts are needed to reduce both the supply of tobacco (regulation around sales and distribution of tobacco products) and demand (tobacco promotions) [8]. In line with the WHO FCTC efforts to reduce tobacco use in the Pacific region have been oriented towards public policy approaches including legislation to control the sale of tobacco to minors, extending environments, taxation and banning tobacco advertising, promotion and sponsorship [9, 10]. However, progress is often slow due to limited resources and competing health demands (e.g. disease outbreaks) [11]. A review of tobacco advertising bans, conducted in eight of the 14 Pacific Islands Countries (PICs) who are signatories to the WHO FCTC, identified that only three (of the eight reviewed) had evidence of implementing a comprehensive tobacco advertising and promotion ban [12].

Although there is considerable information describing the various risk factors and pathways to smoking among adolescents in high income settings, including New Zealand, the same does not exist for adolescents in Pacific countries. Survey data from the Global Youth Tobacco Survey (GYTS) provides some bench-mark prevalence data on youth smoking prevalence, but adds little to a deeper appreciation of the key drivers or mechanisms of uptake. The Samoa GYTS was last conducted in 2007 when 23% of youth aged 13-15years were reported to be current smokers [13], over 60% spent time with people who smoke, including smoking in their home (59%). Awareness of pro-smoking advertisements in media was also common among the GYTS sample, (70% saw a pro-smoking advertisement or media in the past 30 days). At the time of research, the 2013 WHO STEPs data was not available which would have provided a current baseline profile of tobacco use among young Samoans aged over 25 years. Data on the social and cultural influences to uptake is routinely collected via this tool. This information is valuable for generating persuasive and culturally relevant tobacco-free messages, as part of a wider tobacco control programme.

Media is well recognized as a key factor shaping young peoples’ social and psychological development [14–17]. Over the past decade, extensive research has been undertaken to measure and understand the impact of media portrayals of tobacco use on young people’s tobacco related attitudes and behaviours [18–22]. Most notably, the bulk of these studies have been conducted within high income settings where access to media, parental expectations and rules around media use and interpretations are likely to reflect the dominant cultural values. In essence, the most pervasive findings from international studies points to media being a significant informant in the shaping of perceived norms about tobacco use [23]. Increasingly research is focusing on the type of media being consumed by young people. Access to digital media, in particular cell phones and the internet, has radically increased in the Pacific and is likely to be focal point for entertainment and information for young people [15, 23–25]. It is a shift that has not escaped the attention of the tobacco industry. The internet or and social networking sites specifically, are invaluable for circumventing tobacco promotion restrictions [23, 26].

The role of media as an influence on attitudes towards tobacco use, among Pacific youth has been largely overlooked, although the relationship between media use and familial values is a possible mediating factor [27, 28]. In earlier work we identified that smoking imagery in film was perceived as an accurate reflection of real life smoking, albeit distorted in terms of actual smoking rates [19]. Among New Zealand based Pacific students, tobacco imagery was predominantly viewed in relation to expression of style and self-image (e.g. attractive, sexy) [29]. In other work, we identified that adolescents who lived in homes where there were clear non-smoking expectations were less likely to view smoking in media in characteristically positive terms [30]. Parental influence is therefore critical, but not only via what they practice, but also what they expect from their family. We are curious to identify whether these issues play out in Samoa, where family plays a central role in social development of young people [27].

Consistent with current international marketing strategy employed by the tobacco companies, the most viable new markets are those based in low and middle income countries, particularly those with emerging economies with weaker tobacco control regulations. The Western Pacific Region is one of the fastest growing markets for tobacco products, driven in part by the strengthened Chinese economy and resultant growing middle class [31]. Although the Pacific Islands represent a relatively small market for the tobacco industry, the young, mobile and increasingly media savvy populations present a viable market [25]. Adolescent and young adult years are critically important for initiation of tobacco use [32], a fact well exploited by the tobacco industry [33]. Our research is focused on this age group as they are typically early adopters of new media and are therefore vulnerable to influence by the tobacco industry – a factor that has potential for counter-marketing approaches to reduce uptake. Emerging data (mostly produced by regional economic agencies) indicates a consistent increase in access to digital media across the Pacific Islands region, with most dramatic shifts in media and communications delivered via online and mobile devices [24, 34]. Exploring technology and media transformation in respect social change is now highly relevant, especially within the context of Samoa, where traditional family and Christian values are central to social order [35, 36].

This qualitative study examines how young people in Samoa describe tobacco use in Samoa and the influential factors they attribute to current tobacco use among their peers. In-depth qualitative data are fundamental when considering priorities for tobacco control messages and measures, such as the provision of tobacco cessation support in a low resource setting where family and culture are pivotal to change.

## Methods

A qualitative method was undertaken whereby a series of 30 in-depth semi-structured interviews were conducted with high school aged students (males and female, aged 16 and 17 years) from two schools in Apia, Samoa, during April and May 2013. In depth interviews were selected as the primary method due to the potentially sensitive nature of the topic and to avoid group-mediated responses that may distract from participants idiosyncratic views on tobacco use and the media.

### Procedure

Following the receipt of ethics approval from the University of Auckland Human Participants Ethics Committee (reference UOA/9022) and the Ministry of Health, Samoa, two secondary schools in Apia were invited to participate in the study. The schools were selected on the basis that they represented both a high and a medium to lower socio-economic area and were willing to participate and able to consent to participate in the study. Schools were invited via a letter and subsequently followed with a phone call. The Ministry of Education, Sports and Culture (MESC) were consulted and permission was also sought. The interviewer, (HT), who is Samoan and based in Apia, visited each school to discuss the research purpose and method of data collection and explained the process for invitation of students. It was confirmed that up to 10 students (male and females) would be interviewed from each of the two schools invited to participate. Students provided informed consent from their parents in order to participate in the interviews; the confidentiality and voluntary nature of participation was explained and reiterated again at the time of interview. Visits were scheduled for each school to undertake the interviews during class time. All students who provided consent to participate in the study were interviewed during class time (but in a quiet location on school grounds). Interviews conducted in either English or Samoan, were audio-recorded at the time of interview and later transcribed verbatim. Several interviews were conducted in the Samoan language and were subsequently transcribed in English for analysis.

Each student spent up to 30 minutes with the researcher discussing the pre-defined topics before returning to class. The topics covered in the interviews included: background perceptions on tobacco use (including chewing tobacco) in Samoa, cultural and social norms around tobacco use, personal media use (access to media on a regular basis, including social media, patterns around media use, including where, with whom and how often media used, and preferences for different media platforms (e.g. television, radio, internet) and types of programmes/content), and awareness of tobacco imagery in media (mainstream and digital, social media). Information on current smoking status was not formally collected.

Interview transcripts were provided to each of the three researchers to review and read thoroughly to generate a first level of coding for the preliminary analysis. Further analysis was conducted to the point of theoretical saturation, whereby there were no advances on the established themes generated in the second tier of analysis [37]. We did not use a qualitative analysis software package for analysis as it was not considered necessary with the collaborative process of analysis conducted between the three researchers. Discussions were held via Skype to agree upon coding development and final analysis. In the presentation of our qualitative research, we have adhered to the RATS guidelines for reporting qualitative analysis.

## Results

In total 30 interviews were conducted; 19 females and 11 males from two schools. Analysis of the textual data are presented according to the dominant themes: *Smoking as a reflection of family standing on morality; Tobacco sales: a family business; Tobacco control: ambivalence about impact on smoking; Perceived resilience against effects of advertising, graphic warnings and other tobacco control media; Influence and use of entertainment and online social media*. Strikingly, there was a general lack of understanding about the influence media has on tobacco use opinions, attitudes and behaviour.

### Smoking as a reflection of family standing on morality

There was a great deal of consistency across all interviewees, that family and peers were the primary determinant of smoking uptake among young people in Samoa. As stated by one participant; “*the adults have to set the example first…I would remember growing up, all I would hear is, it’s bad for you, it’s bad for you, don’t go there. You never get why…*” Equally, coming from a large family that “*wasn’t strict enough*” or failed to provide strong moral guidance, was viewed as a possible reason some young people in Samoa started smoking.

Tobacco use was framed as a moral and a social issue by many of those interviewed. Being “*raised well*” in a Christian family was seen as being protective against smoking. One student felt her own family attitudes protected her, *“like for me, I come from a family that are really against it and I still have that foundation not to go there”.* But this sentiment is in direct conflict with her other views about smoking uptake, *“the fact knowing that it’s not good makes them want to do it more. It’s cool and it’s edgy!”* Non-smoking participants reported that they modelled their behaviour on family expectations. They viewed peers who smoked to come from families that lacked moral standing. To a lesser extent, friends were also mentioned as a possible driver to tobacco use; citing the pressure to ‘fit in’ in social situations. One student indicated that she felt vulnerable to becoming a smoker as her best friend smokes.

The majority of students we interviewed were familiar with the health risks associated with smoking. Several participants were less clear about the effect of tobacco use physiologically: “*That’s my question too – its only smoke, the smoke that comes from burning…but my dad used to smoke before getting into church. He said he did it to decrease pressure. If he smoked he said he would be satisfied. So that makes me confused*”. These questions may reflect awareness created by a recent mass media campaign that highlighted the health risks of tobacco use. The majority of participants however, expressed versions of *“disgust”* regarding tobacco use; one suggested that teenagers who did smoke were *“ignorant”* of the consequences of tobacco use. Addiction was seldom mentioned with only a few framing smoking as a *“bad habit”* and the personal choice of the smoker.

Smoking by older people was very much seen as a matter of individual choice, with one interviewee stating that, “*yeah, I am just against it for the younger generation but for the older ones, it’s their choice*”. The impact of taking an individual responsibility approach to smoking on others smoking was raised. The choice to smoke was viewed by several participants as also having a negative impact on the country, with several participants articulating that smoking rates and the health effects of smoking were huge concerns for the future of Samoa.

### Tobacco sales: a family business

Several participants interviewed spoke about how their own families owned a small shop and sold tobacco. There were mixed beliefs on the role of a local retailer in supplying tobacco. One student described tobacco as a “*popular and quick selling*” item that her parents simply had to provide and that it was up to customers if they purchased the product. Another noted the importance of tobacco sales in her family store; “*Well, we do have a shop and that’s the most important product, that’s the fastest selling in the shop on a daily basis. So we are 4 cartons a day*”. Others reported their experience of local businesses willingly selling tobacco products to youth with little regard to the health consequences, for example: “*we have a shop at [village name], even the young people they [buy] it, one or two*”. Selling cigarettes to youth was justified in some cases, as it was known that the purchase was for their smoking parents.

### Perception of tobacco control in Samoa

Despite articulating their concern about the potential impact of smoking on the future of Samoa, when asked about the importance of tobacco control polices such as on-pack graphic health warnings and banning tobacco advertising few agreed these were likely to be effective initiatives. Most of those interviewed were familiar with graphic health warnings, with several stating they found these images disturbing or shocking and one student commenting that the images made her think smokers were *“committing suicide.”* Despite holding negative reactions to the health warnings, students generally did not feel that the warnings were likely to motivate smokers to quit. There was some agreement that perhaps for those who *“wanted to quit”* the warnings may be useful, but because people continued to smoke in spite of the warnings this was presented as evidence that they really had no effect. There was perhaps a lack of appreciation that health warnings are government mandated, with some students confused as to why local shops would both sell tobacco and warn smokers about the dangers of using the product. Comments such as “*I don’t really think it is effective because we are buying it in the first place. Since we’re like selling it in the first place. I mean it is effective to those who rarely smoke or only smoke then they’re depressed and stuff*”, reflect the level of ambivalence felt about anti-smoking efforts, especially those displayed on packs or in stores which sell tobacco.

Concern about the broader impact of tobacco on Samoa, as a country, was also expressed by several participants and appeared to be somewhat protective in preventing tobacco use; “*it has to be looked at; it is a threat to people’s lives but also affects the economy of the country*” and “*I think it [buying smokes] is not really good for Samoa itself*”. Yet, there was not strong support, or at least appreciation of the role of tobacco control policies such as graphic health warnings or increasing the price of tobacco increases in taxation to reduce consumption. Ambivalence about the importance of regulating tobacco advertising combined with the views that smoking is primarily due to family influences suggests that students do not believe tobacco use is a public issue that is amenable to change through policy and regulation. Smoking was framed as either an individual shortcoming or legitimate adult behaviour and as such very little could be done to effect change.

The display of tobacco products at retail was not generally viewed as a form of tobacco promotion. One student who worked in her family’s shop did not believe the business promoted the tobacco products it sold, *“no, we pretty much just sell it but we don’t advertise”.* When asked about any specific promotions and advertisements they had seen at retail, one student described some posters she had seen, “*in front of the shops, like for example, Pall Mall, there are big posters in front of the shops and then the stock is placed in front of it*”. But again, the idea that this influenced people to smoke was not generally held, and that smoking could be explained primarily by personal preference, *“Some smoke, some don’t, is up to them”.*

### Perceived resilience against effects of advertising, graphic warnings and other tobacco control media

There was similar ambivalence about the effects of tobacco advertising and the need to ban tobacco advertising as a way of reducing smoking rates. While there was some understanding that advertising played a role in helping to make something that might be *“bad”* seem like it was *“good”* there was a lack of understating about how advertising influences consumers decisions. Students were unsure why smoking was *“cool”* with one describing it *as “starting out that way and it stuck”.* Advertising was generally only described as that found in traditional mass media channels, such as television ads, billboards or posters. Tobacco displays at retail, branding on packages, or pro-smoking content online were not viewed as forms of advertising. Being unaffected by advertising messaging appears to be a source of cultural pride, “*I mean we’re Samoans, we don’t care about advertising*.” Another student thought the impact on adult smokers would be minimal; *“Well, for me, I don’t think it works because for my parents, they don’t actually read it, they smoke it”*. Alternatively, graphic warnings were thought to have a greater effect on kids who had not started to smoke, as suggested by one participant *“[seeing the ads] would have struck something in my head, not to smoke, but I think for the true smoker, they wouldn’t really care*”.

### Influence and use of entertainment and online social media

Pro-smoking imagery in entertainment and online social media was not universally seen as important or influential. Parental regulation of media use was still common among the high school student with some referring to families practicing Christianity as the reason for restriction. One participant commented on the profound impact of the church and family on media use “*Well, I grew up in a Christian family, so I am not interested in those kinds of things, because I am not allowed*…*Plus technology nowadays is very fast, it is another reason my dad does not allow us on the Internet”*. Exposure to inappropriate or amoral content in media was one of main reasons parents’ restricted use. Cost of accessing the internet, which is less accessible and more costly than cell phones, was also cited as a reason for restriction.

Some students agreed that seeing their favourite celebrities smoking could influence very young children to smoke. Other students were sceptical that this type of smoking imagery had any effect, *“I don’t really think [the] Internet can really affect the kids.”* Another perspective suggested that that direct advertising in media was likely to be less influential than “*ads and background of people – that’s when I see, I saw Simon [from American Idol) smokin*g”. Students did generally agree that smoking imagery in movies and online was common. Again, this type of imagery was not described as a form of advertising and no student suggested that the tobacco industry could be using entertainment or online social media to promote its products. A few students said that they had seen actual tobacco advertisements online, but were unable to give specific examples. Several students said that they had seen photos of their peers smoking being shared on social media sites, like Facebook: “*Some kid, when you go through their photos smoking… [are] holding cigarettes, drinks, alcohol, so it’s really common. You can tell who smokes when you see their photos…”.* Facebook was considered a more authentic representation of actual smoking; “*Well, movies, people are making it up, people are acting out so it is not really happening. Somehow, but then Facebook is what’s really happening daily”.*

Social media use was relatively common among the students interviewed. Facebook was the most popular online platform used by students, with one student suggesting that the “*whole school*” had accounts on the site. Many students had their own mobile phones and were able to access Facebook on their phones. In this respect, media use was not as easily regulated by parents. Although, this was not a universal experience with some students banned by their parents from opening a Facebook account and were not permitted to access their account from their phone. Again, the tension between familial values and morality and media use, especially new media, was evident.

## Discussion and conclusions

Our study describes young Samoan student’s perceptions of the social and media drivers to tobacco use in Samoa. Although the interviews were tailored to explore the role of new media in forming perceptions of tobacco use, it was apparent that the primary drivers to expectations remain fundamentally about relationships with family and Samoan cultural values. We noted a pervasive and positive Samoa pride expressed throughout the interviews. Samoa as their country, family as the core foundation to society was evidenced in our discussion about drivers to tobacco use. The sale of tobacco in the local shops was mentioned as a core source of income for some families which raises the issue of loyalty and values for some participants. With high rates of tobacco use among adults in Samoa, there is a strong incentive to introduce measures to reduce tobacco use among this group. Youth smoking uptake is likely to be influence by a range of factors, as noted in other international studies, but Samoan youth are highly connected to family and to their country to an extent not observed in Western societies. Targeting adult and youth current tobacco use through increased knowledge of and access to tobacco cessation support, will contribute to reducing uptake among young people in Samoa. This could work well alongside on-going plans to implement and strengthen WHO FCTC aligned public policy measures.

Students were not overly critical of media constructions and showed little awareness that online social media could be used by corporations like the tobacco industry, for purposes of marketing and promotion. Increasing media literacy, particularly in the face of increasing access to online channels and the relaxing of parental controls of media use, is a potentially important focus for health promoters and educators, particularly in the context of increased media usage and marketing. Previous research identified that young people are reluctant to attribute behavioural influence to external sources such as advertising or other media, whereas they are more likely to accept family or peer influence [30, 38, 39].

Tobacco use contributes to undermining efforts to reduce the escalating rates of NCD in the Pacific region [40]. Tobacco control policies aimed at reducing uptake and increasing quit behaviours are gradually being introduced in line with the WHO FCTC, of which 14 PICTS are signatories [9]. Although Samoa represents a small portion of total product sales in the region, it nonetheless is strategically well placed as a tourist location and the home for a predominantly youthful and mobile population. With the Trans Pacific Partnership (TPP) agreements in negotiation and no resolution as to where to place tobacco, it is likely that tobacco control in the region will remain a challenge [41]. Over the past few years the Pacific region has increased access to telecommunications due to the “opening of new markets, communication tools and greater diversification of markets and services” [42]. The transformative impact of globalisation on life in Samoa has undoubtedly altered patterns of media consumption [43–45]. Yet, MacPherson and MacPherson, taking a fa’a-Samoa perspective, a Samoa-centric and culturally determined view on transformative (or transnationalism) processes in Samoa, suggest that New Zealand, Australia and the United States, are “sites of transnational, triangular and circular exchange” and therefore the cultural practices of transnationalism, (as form of globalisation), has been significant facet of fa’a-Samoan life for some time. However, access to digital media, such as telecommunications and the Internet has further accelerated this evolution.

Deference to adults remains a fundamental facet of family and social order in Samoan society, albeit one that is constantly under challenge with shifting of traditional values and norms [46]. Familial and village hierarchies, defined in terms of the Matai structure, the church and the extended familial lines remain powerful influences and potential avenues for influencing public health policy in Samoa [47]. Research on Samoan adolescent substance use suggests that alcohol and cigarettes in particular were associated with the (desired) transition to adulthood, a reflection of being mature and even modern – or “cool” just as we found in our study [48]. This is consistent with international evidence on the drivers of smoking uptake, evidence known to be well utilised by the tobacco industry.

Possible limitations of our study include that interviews were conducted in the local Samoan language and were later transcribed by the researcher (HT) into English for analysis. This may have resulted in the loss of data integrity in some sections and the nuances captured via indigenous language and non-verbal expression. Similarly, the interviewer was a former school teacher and may have been known by some students, which could have precluded a full and open discussion of the issues presented, but we minimized this by not asking directly about student smoking status. Samoan cultural values of respect for elders, including in conversation, may have limited the depth of discussion between the interviewer and students. In addition, although we did not specifically recruit non-smokers, the majority of participants either chose not to indicate their smoking status or were non-smokers. It therefore begs the question of whether our results would be significantly different should we have had interviewed smokers. This would have added considerable value to our research.

The level of enthusiasm for social media is an important consideration in the development of more innovative modes of delivery for tobacco control communication. With the evolution of broadband connectivity the future for social media as a mechanism for exchange will be fundamental to health. It can no longer be assumed that online media exposure in low and middle-income countries is of minimal importance [49]. It is essential to understand how the acceleration in smart phone ownership and availability of mobile and internet networks could be impacting on tobacco use. Given that Facebook was nominated as a popular social media platform, experimenting with low-cost interventions to mobilise and increase support for tobacco control policy could be a valuable and low-risk investment. This may prove to be particularly important in shifting perceptions that tobacco use is an individual or family responsibility to one that demands political action and a whole of society approach. Media literacy programmes run through schools have been advocated as a potential counter measure to the rise of media as an influence in young people’s lives. Evidence from a randomised controlled trial of media literacy programme on reducing tobacco use found that schools who implemented this training increased students awareness of how media influences behaviours around tobacco use [50]. Raising awareness of the rise of the tobacco industry role in promoting tobacco use was found to be effective in trials of the US “Truth campaign”. These strategies tap into young peoples’ sense of justice and fairness and patriotism [51, 52]. Our participants may well be motivated by evidence of the impact of tobacco industry profits on Samoa’s health and future development.

At the present time in Samoa, a mass media campaign is underway that is focused on raising awareness of the health effects of tobacco use. It is too early to determine the impact of this intervention, but evidence from other settings determine the importance of a broad approach to tobacco control, whereby mass media sits alongside public policy interventions (e.g. increasing smoke free environments, raising taxes on tobacco) and tobacco cessation support. The findings from this work provide insights how youth in Samoa perceive the drivers to tobacco use and recognises that young people are highly conscious of social change and are themselves, influential to social change. Given the perceived influence of parental smoking on youth uptake in Samoa, efforts to reduce adult smoking would be a worthwhile intervention at a policy and practice level. Targeted inventions designed to promoting cessation are now a recognised priority for tobacco control in Samoa and work is underway to progress these plans in alignment with the WHO FCTC Article 14 [9, 10].
